# Effect of Various Irrigant Activation Methods and Its Penetration in the Apical Third of Root Canal— *In Vitro* Study

**DOI:** 10.1055/s-0041-1742122

**Published:** 2022-02-21

**Authors:** Delphine Pricilla Antony S., Pradeep Solete, Ganesh Jeevanandan, Ather Ahmed Syed, Samaher Almahdi, Mohanad Alzhrani, Prabhadevi C. Maganur, Satish Vishwanathaiah

**Affiliations:** 1 Department of Conservative Dentistry and Endodontics, Saveetha Dental College and Hospitals, Saveetha Institute of Medical and Technical Sciences, Saveetha University, Chennai, Tamil Nadu, India; 2 Department of Pediatric and Preventive Dentistry, Saveetha Dental College, Saveetha Institute of Medical and Technical Science, Chennai, Tamil Nadu, India; 3 Division of Pedodontics, Department of Preventive Dental Sciences, College of Dentistry, Jazan University, Jazan, Kingdom of Saudi Arabia; 4 Ministry of Health, Kingdom of Saudi Arabia

**Keywords:** endodontic irrigant, EndoActivator, iohexol dye, profit s3, side-vented needle

## Abstract

**Objective** The objective of this study was to evaluate the irrigant penetration using iohexol dye with four irrigation techniques.

**Methodology** Single-rooted premolars were recently extracted and preserved in physiological saline solution. All the samples were standardized to 16 mm. Standard endodontic access was prepared using endoaccess bur (Dentsply Maillefer, Switzerland). The initial patency was established using #10 k file (Mani, Utsunomiya, Tochigi, Japan) to the working length. The cleaning and shaping were performed using the file system ProFit S3 in the following sequence: P0 (orifice enlarger), PF1 (yellow), PF2 (red) #25, and PF3 (blue) #30. The samples were randomly allocated in concealed opaque envelopes into four groups. This was performed by a trained dentist. Fifteen samples were allocated to one group. The groups were divided as follows: Group A—conventional needle (CN), Group B—side-vented needle (SVN), Group C—manual dynamic agitation (MDA), and Group D—EndoActivator (EA). The radiopaque dye irrigant agitation/activation was performed by one operator to prevent operator bias. Following irrigation using the different techniques, digital radiographs were taken, and the measurement was taken from the apical foramen to the point where the dye had penetrated apically for each tooth and the data were entered into an Excel sheet for all the four groups.

**Results** Comparing the four groups, there was a statistically significant difference among the four groups ( *p* < 0.05), thus, favoring the alternate hypothesis. EA had resulted in better penetration of the irrigant compared with the other three groups ( *p* < 0.05).

**Conclusion** It was evident that irrigant penetration was best achieved with the use of an EA followed by MDA, SVNs, and then the CN when the preparation was done till size 30 (PF3 #30) using ProFit S3 rotary file system.

## Introduction

Disinfection is an integral part of root canal treatment along with cleaning and shaping. The root morphology of teeth varies, and to obtain adequate disinfection root canal, irrigant plays a vital role. Irrigation protocol is followed, which provides for chemical dissolution of the pulp tissue, removal of smear layer, dentin debris/shavings, and mechanical uncoupling of biofilm thereby reducing the number of microorganisms. 1 The activity of the best irrigant is obtained only when it reaches the site of the bacteria within the canal and fulfills its required goal of disinfection. Adequate and effective delivery of the irrigant and activation/agitation of technique play a pivotal role to guarantee adequate quantity is delivered within the root canal and adequately replenished to maintain the desired concentration of the irrigant. The irrigation technique used should bring about a flow property that pushes out the microorganisms, biofilm, and tissue remnants out of the canal 2 in the coronal direction. The activity of the irrigant should be confined to the root canal only. Irrigants used should provide adequate disinfection, and it should not alter the physical and chemical properties of dentin. 3 4

The apical third of the root morphology is complex 5 with numerous lateral canals, apical delta, and apical ramifications. 6 Disinfection of these areas cannot be adequately obtained only with cleaning and shaping. Irrigants play a major role in the disinfection of these areas and provide the success of the root canal treatment. To obtain adequate and effective disinfection of the root canal, the use of irrigation techniques plays a major role. Different methods or techniques are used such as ultrasonic activation, manual agitation techniques, machine-assisted agitation, continuous irrigation, sonic activation, apical negative-pressure irrigation, laser activation, photo-activated disinfection, and ozone. 7

The age-old practice was normal syringe irrigation for the delivery of irrigants. This is the most common and popular method due to its simplicity and ease of use. Various syringe and needle sizes were used based on the clinician's requirement. Syringes of size 5 to 20 mL are commonly used. A wide variety of irrigant delivery needles are used during root canal procedure. 2 8 9 Needles are usually made of stainless steel, but NiTi 10 and recently, plastic is also used which increases the flexibility and ease of use in curved canals. 2 The diameter of the needle is represented in “gauge” units, 10 11 the higher the unit value, the finer the needle. Larger size needles were used previously, and a lesser quantity of irrigant was delivered with the restricted flow to the apical region. In recent times, the delivery of irrigant to the total length of the canal is emphasized. The use of smaller needles has obtained recognition. The disadvantage with a smaller needle is that more force is required to deliver the same flow rate, and a minor decrease in the diameter of the needle will lead to an increase in the force required. 12

Irrigation needles fall under two categories: open-ended and closed-ended. Open-ended needles devise an intense jet of irrigant which is directed apically. 10 13 The factors that influence the penetration of the irrigant are apical size, taper, and the flow rate of the irrigant. 13 14 15 16 Closed-ended needles create a low-intensity jet 14 that channels the irrigant toward the root canal walls.

Manual dynamic agitation (MDA) is not a piece of equipment that is used, rather a file (instrument), gutta-percha cone/point, or endodontic brushes are agitated within the root canal along with the irrigant. 17 This method causes displacement of the endodontic irrigant solution into the canal coronally, apically, and into the isthmus and uninstrumented fins. 18 After the root canal instrumentation is complete, a gutta-percha point/cone that matches or closely matches the apical preparation is selected 19 and its tug back or tight fit is verified for the effectiveness of this agitation process. During this agitation process, there can be an extrusion of irrigant through the apical foramen 20 and to counter this effect, the gutta-percha cone/point is trimmed 1 mm at the tip to prevent extrusion. 17

Sonic agitation relies on the transverse oscillation of a tip that is placed within the confines of the root canal to agitate the irrigant at lower frequencies 160 to 190 Hz for the EndoActivator (EA) (Dentsply Sirona, Charlotte, North Carolina, United States) 21 to higher frequencies as high as 6,000 Hz for EDDY (VDW, Munich, Germany). 22 The agitation process is performed after the delivery of the irrigant using a syringe and needle.

The aim of the current study was to evaluate the depth of penetration of four irrigation techniques using a radiopaque contrast dye. The null hypothesis was that there was no significant difference among the four irrigation techniques used.

## Materials and Methods

Single-rooted premolars were recently extracted and preserved in physiological saline solution. The teeth were examined using a radiograph to identify any canal deviations and calcification of canals. Such teeth were eliminated from the study sample. Sixty teeth samples were randomly divided into four groups and used for evaluating the irrigation technique. The root curvature was standardized according to Schneider's technique to 20 to 40 degrees. The crowns were decoronated to a length of 16 mm to maintain uniformity of the teeth and were mounted on a wax block. The apical foramen of the roots was sealed using wax to simulate closed system.

Standard endodontic access was prepared using endoaccess bur (Dentsply Maillefer, Switzerland). The initial patency was established using #10 k file (Mani, Utsunomiya, Tochigi, Japan) to the Working Length (WL). The cleaning and shaping were performed using the file system ProFit S3 in the following sequence: P0 (orifice enlarger), PF1 (yellow), PF2 (red) #25, and PF3 (blue) #30. 23 24 The samples were randomly allocated in concealed opaque envelopes into four groups. This was performed by a trained dentist. Fifteen samples were allocated to one group. The groups were divided as follows: Group A—conventional needle (CN), Group B—side-vented needle (SVN), Group C—MDA, and Group D—EA.

### Preparation of dye

The radiopaque iohexol dye was mixed with 5.25% sodium hypochlorite by a trained pharmacologist in the proportion of 45:55 to obtain a radiopaque irrigating solution with a density (1.0848 g/mL) and surface tension (75.60 dyne/cm) similar to NaOCl 2 to 5%. 25

The radiopaque dye irrigant agitation/activation was performed by one operator to prevent operator bias. Following irrigation using the different techniques, digital radiographs were taken, and the measurement was taken from the apical foramen to the point where the dye had penetrated apically for each tooth, and the data were entered into an Excel sheet for all the four groups.

### Statistical Analysis

The data obtained were entered into an Excel sheet and the data were transferred to SPSS software. The statistical analysis was performed using the SPSS software version 23.0. Descriptive statistics was performed to calculate the mean and standard deviation. One-way analysis of variance and post hoc Tukey's test were done to compare significance among the four groups.

## Results

**Fig. 1:**
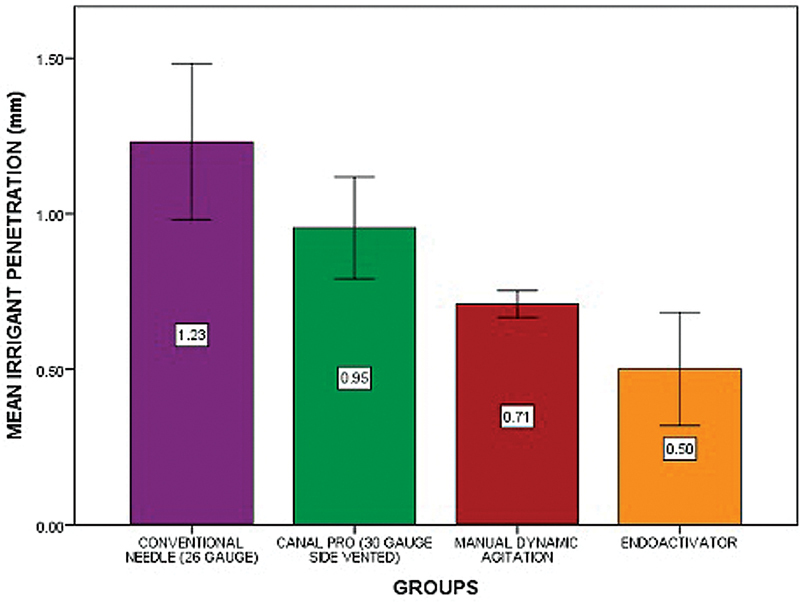
The bar chart depicts the digital measurement from the apical foramen to the point where the dye has penetrated in the canal of conventional needle (purple), side-vented needle (green), manual dynamic agitation (red), and EndoActivator (yellow). *X* -axis represents the measurement of the distance between the dye and the apical foramen. *Y* -axis represents the four groups of irrigation techniques. There was a statistically significant difference among the four groups at *p* < 0.05.

Comparing the four groups, there was a statistically significant difference among the four groups ( *p* < 0.05) ( Fig. 1 ). The mean and standard deviation of the groups are as follows, Group A (CN)— 1.2313 0.25145, Group B (SVN)—0.9547 0.16444, Group C (MDA)—0.70930.04399, and Group D (EA)— 0.5013 0.18185, respectively, on group-wise comparison, EA was found to be significant compared with other three groups ( *p* < 0.05), MDA, SVN, and CN, thus favoring the alternate hypothesis. The EA group had a mean and standard deviation of 0.5013 ± 0.18185 with a *p* < 0.05. Thus, the replacement of irrigant was best achieved with the use of EA.

## Discussion

Disinfection in endodontics is achieved by the combined effect of root canal irrigant and endodontic files. Irrigants have two actions, mainly antibacterial action and decalcifying action. 26 Endodontic irrigant as such does not completely disinfect or reduce the microbial load. Both hand and rotary instruments are constantly improved, 27 yet more than 40% of the root canal surface remain uninstrumented. 28 It requires a better delivery system and agitation to bring about the best of the irrigant used in achieving disinfection of the root canal. The aim of a delivery system is to transfer or transport the required quantity of the irrigant into the main canal, and agitation or activation carries this irrigant further into the canal system and enhances the disinfection process. 2 The effectiveness of endodontic irrigation was the depth of penetration of the needle and the distribution of the irrigant beyond the tip of the needle which was much less than expected according to Chow. 28 29

In the current study, the patency was established to size #10 using a K-file, and then cleaning and shaping were achieved using the rotary file system ProFit S3. The canals were prepared till the size PF3 #30 with copious irrigation. The teeth were then randomly divided into four groups. Group A—CN (26-gauge needle), Group B—CanalPro—30-gauge needle, Group C—MDA, and Group D—EA. During instrumentation, the radiopaque irrigating solution was delivered passively according to the respective group technique, and irrigant penetration depth was measured using digital radiographs in the conventional group. The measurement was done using a digital–analog scale from coronal to apical direction.

In the current study, there was a significant difference among the four groups in the dye penetration to the apical region. The CN of 26 gauge has a metric size of 0.45 mm and an external diameter of 0.440 to 0.470 mm. These open-ended needles generate an intense jet of irrigant which penetrates apically. 10 13 The depth of penetration of irrigant depends on the apical size and taper of the root canal to which it has been prepared and the flow rate of irrigant. 10 13 15 16 An increase in the taper size leads to better penetration of the irrigant and subsequent flushing out of debris and removal of the smear layer. Khademi et al stated that apical instrumentation to a #30 size file with 0.06 coronal taper is effective for the removal of debris and smear layer from the apical portion of root canals. 28 30 In this study, it was found that an increase in root canal taper that is, #30 resulted in deeper penetration of the needle and enhanced the irrigant replacement. It was found the penetration of dye was less with a 26-gauge needle compared with SVN.

The external diameter of the 30-gauge needle is 0.3 mm which corresponds to 30 size instruments. In this study, the flat open-ended needles were placed 2 mm short of WL and SVNs 1 mm short or where the needle first binds to the canal. In an *in vitro* study done by Boutsioukis et al, 10 it was reported that the SVN achieved irrigant replacement to the WL only at the 1-mm position, whereas the open-ended flat needle was able to achieve complete replacement even when positioned at 2 mm short of the WL. A study by Srirekha et al stated that increased preparation of the canal and the use of flat-ended needles and SVNs had no significant difference in irrigant penetration 28 which is contradicting the current study.

MDA helps the distribution of the irrigant and its exchange within the root canal and enhances the effectiveness of the irrigant solution used. In this process, the irrigant present in the canal reaches the root apex and disrupts the vapor lock. 31 It creates an increased intracanal pressure change within the canal during the forward and backward movement of the gutta-percha cone within the canal which creates turbulences that enhance the diffusion of shear stresses. 32 A previous study showed the risk of apical extrusion during MDA, 33 and this can be strongly avoided by the agitation of the gutta-percha cone 1 mm short of the WL. 32 In the current study, MDA achieved better penetration of the irrigant compared with 26-gauge CN and 30-gauge SVN.

**Fig. 2:**
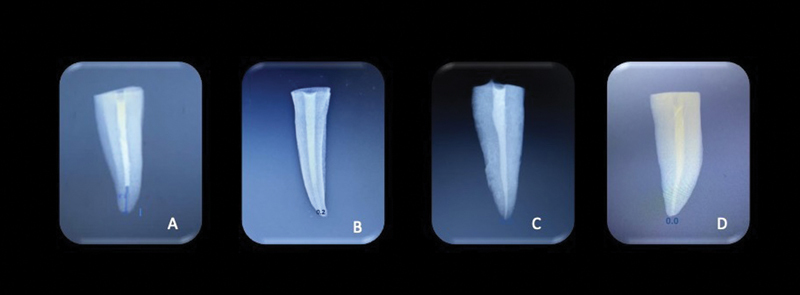
Digital radiographs of the teeth with dye penetration. ( **A** ) Conventional needle 26 gauge, ( **B** ) side-vented needle, ( **C** ) manual dynamic agitation, and ( **D** ) EndoActivator.

Subsonic activation is the third most preferred technique following syringe irrigation and ultrasonic activation. 34 35 For group EA, the irrigant delivery protocol was followed as stated by Ramamoorthi et al. 36 In the current study, EA which comes under subsonic activation had better penetration of the dye ( Fig. 2 ), which implies that more irrigant solutions had been in contact with the canal walls. Kanter et al reported that EA aided in better removal of debris in lateral canals. 37 It was stated by Bago et al that EA had a superior effect compared with needle irrigation. 38 Sato et al stated that effectiveness of the irrigant is influenced by the quantity, temperature, and its interaction with the agents. 39 Iandolo et al in 2020 stated that irrigant activation enhances the efficacy of irrigants. 40 Iandolo et al in 2019 stated that endodontic irrigant activation aids in flushing out of debris and it helps eliminating bacterial load from the root canal. 41

In this study, it was evident that the EA performed better than the other three groups, and in the order, EA, MDA, SVN, followed by the CN.

## Conclusion

Within the limitations of the study, it was evident that irrigant penetration was best achieved with the use of an EA followed by MDA, SVNs, and then CN. The current study being an *in vitro* study needs more clinical trials to substantiate the obtained results in the future.
